# Supplementary material for: Cereblon Deficiency Contributes to the Development of Elastase-Induced Emphysema by Enhancing NF-κB Activation
Source: Antioxidants (Basel). 2022 Oct 4;11(10):1980. doi: 10.3390/antiox11101980 (PMC9598989; doi:10.3390/antiox11101980)
Supplement: Supplementary file 1 [file antioxidants-11-01980-s001.zip › antioxidants-1914816-Supplementary.pdf]

Supplementary Materials and Methods

Reagents

Antibodies used for protein detection were anti-LC3B, anti-caspase-3, and anti-poly (ADP-ribose) polymerase (PARP) antibodies (Cell Signaling Technology, Danvers, MA, USA); anti-p21, and anti-p27 antibodies (Santa Cruz Biotechnology Inc., Santa Cruz, CA, USA).

Flow cytometry

Cells were incubated with anti-MARCO-fluorescein isothiocynate (FITC), anti-SR-A1-phycoerythrin (PE), anti-IgG FITC, or anti-IgG PE in 200 μl incubation buffer for 45 min. Unreacted antibodies were removed. Cell-associated FITC/or PE-conjugated antibodies were analyzed by flow cytometry using a FACSCalibur or a FACSCanto™ flow cytometer (BD Biosciences, San Jose, CA)

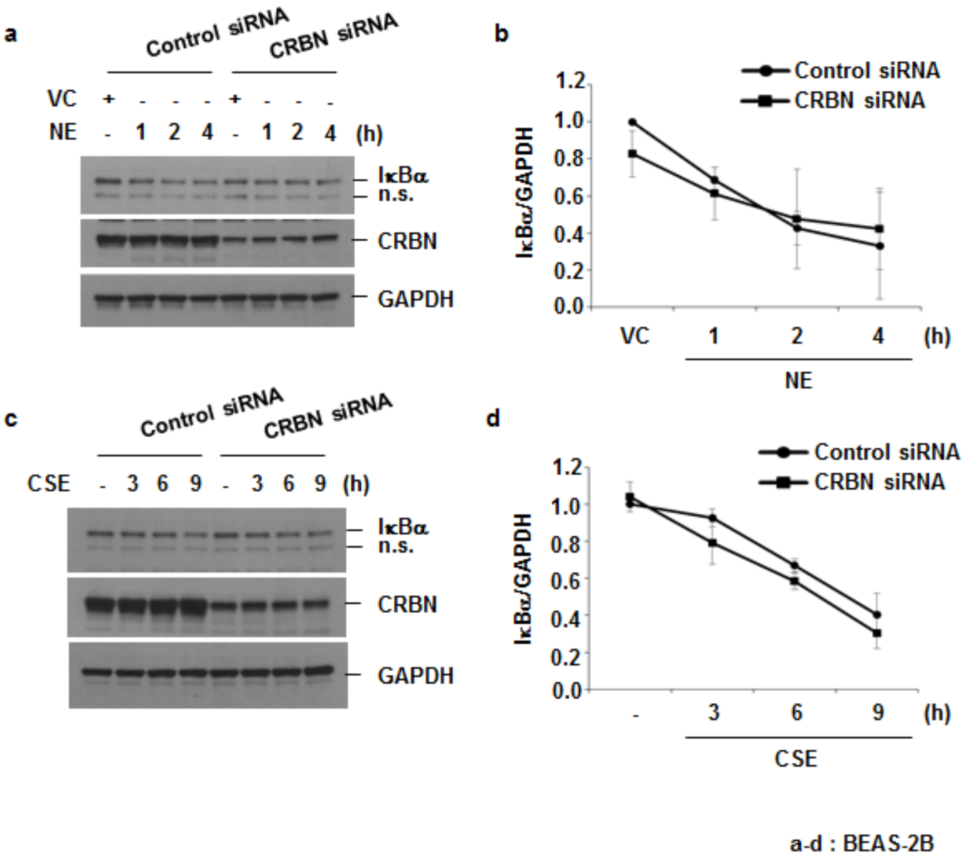

Supplementary Figure 1. *Crbn* Knockdown does not affect NE- and CSE-induced degradation of IκBα. BEAS-2B cells were transiently transfected with control siRNA and *Crbn* siRNA. Forty-eight hours after transfection, the cells were treated with VC, NE (1U/ml) (a, b), or CSE (1%) (c, d) for the indicated times. Total cellular extracts were subjected to Western blot analysis for IκBα, CRBN, and GAPDH (a, c). Densitometry analysis using Scion image software for IκBα. Blots were normalized to GAPDH expression (b, d) Data represent the mean ± SD.

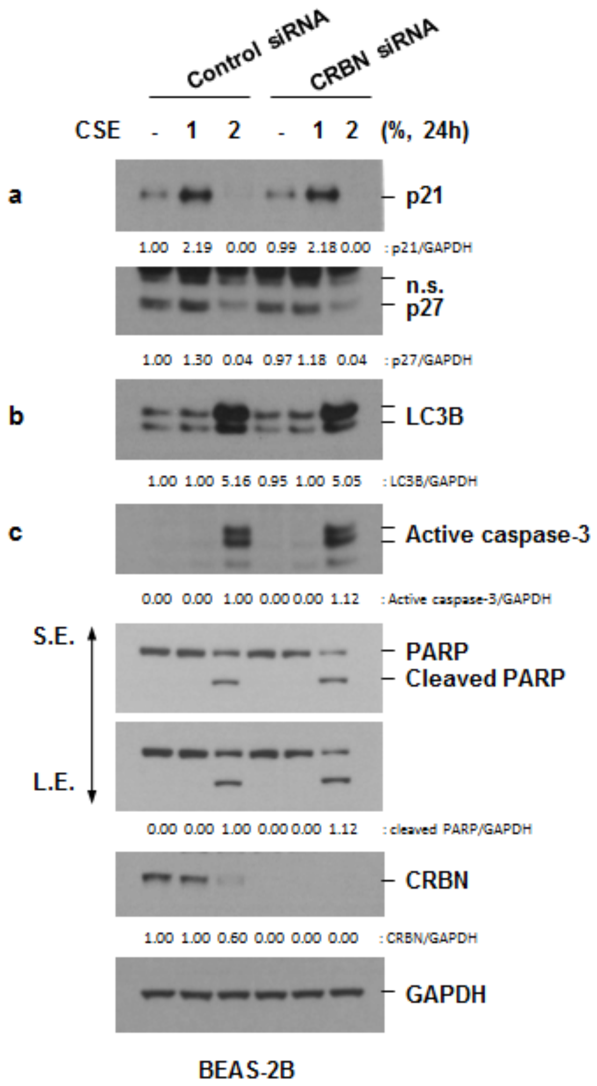

Supplementary Figure 2. *Crbn* Knockdown does not affect CSE-induced aging, activation of autophagy, and apoptosis. (a-c) BEAS-2B cells were transiently transfected with control siRNA and *Crbn* siRNA. Forty-eight hours after transfection, cells were treated with CSE (1, 2%) for 24 h. Total cellular extracts were subjected to Western blot analysis for aging markers (p21, p27) (a), autophagy marker (light chain 3B, LC3B) (b), apoptosis markers (active caspase-3, PARP) (c), and GAPDH. Densitometry analysis using Scion image software for p21, p27, LC3B, active caspase-3, and PARP. Blots were normalized to GAPDH expression. S.E., short exposure; L.E., long exposure

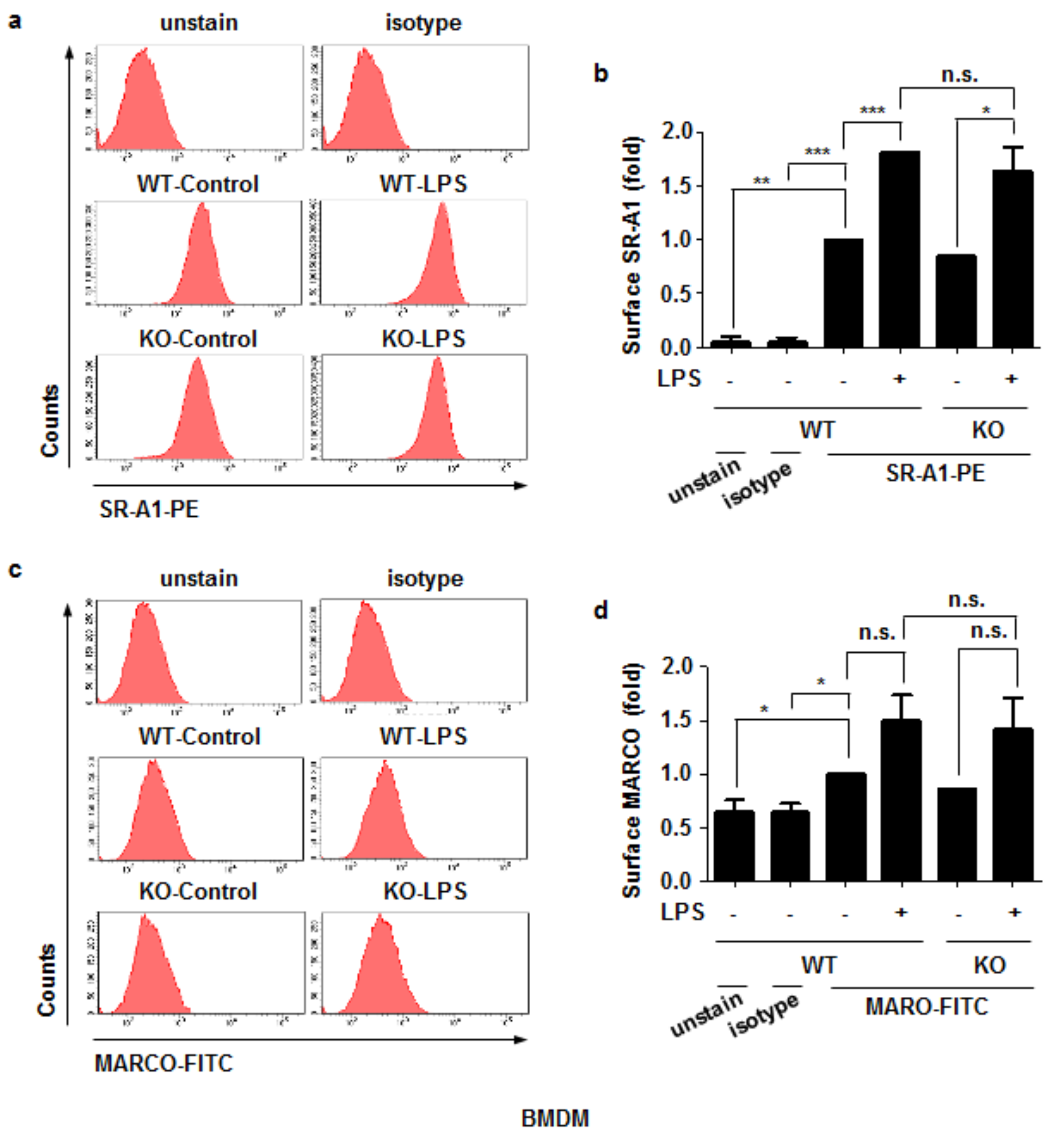

Supplementary Figure 3. *Crbn* KO did not affect the LPS-induced surface expression levels of scavenger receptors in BMDM. WT and *Crbn* KO BMDM were stimulated with LPS (100ng/mL) for 24 h. Cell surface expression of macrophage scavenger receptor class A 1 (SR-A1) and macrophage receptor with collagenous structure (MARCO) was determined by flow cytometry. Results are shown as mean fluorescence intensity (MFI) histograms (a, c). (b, d) Data represent the mean ± SD of two separate experiments. * $p<0.05$, ** $p<0.01$ and *** $p<0.001$. n.s., non-significant; PE, phycoerythrin; FITC, fluorescein isothiocyanate.

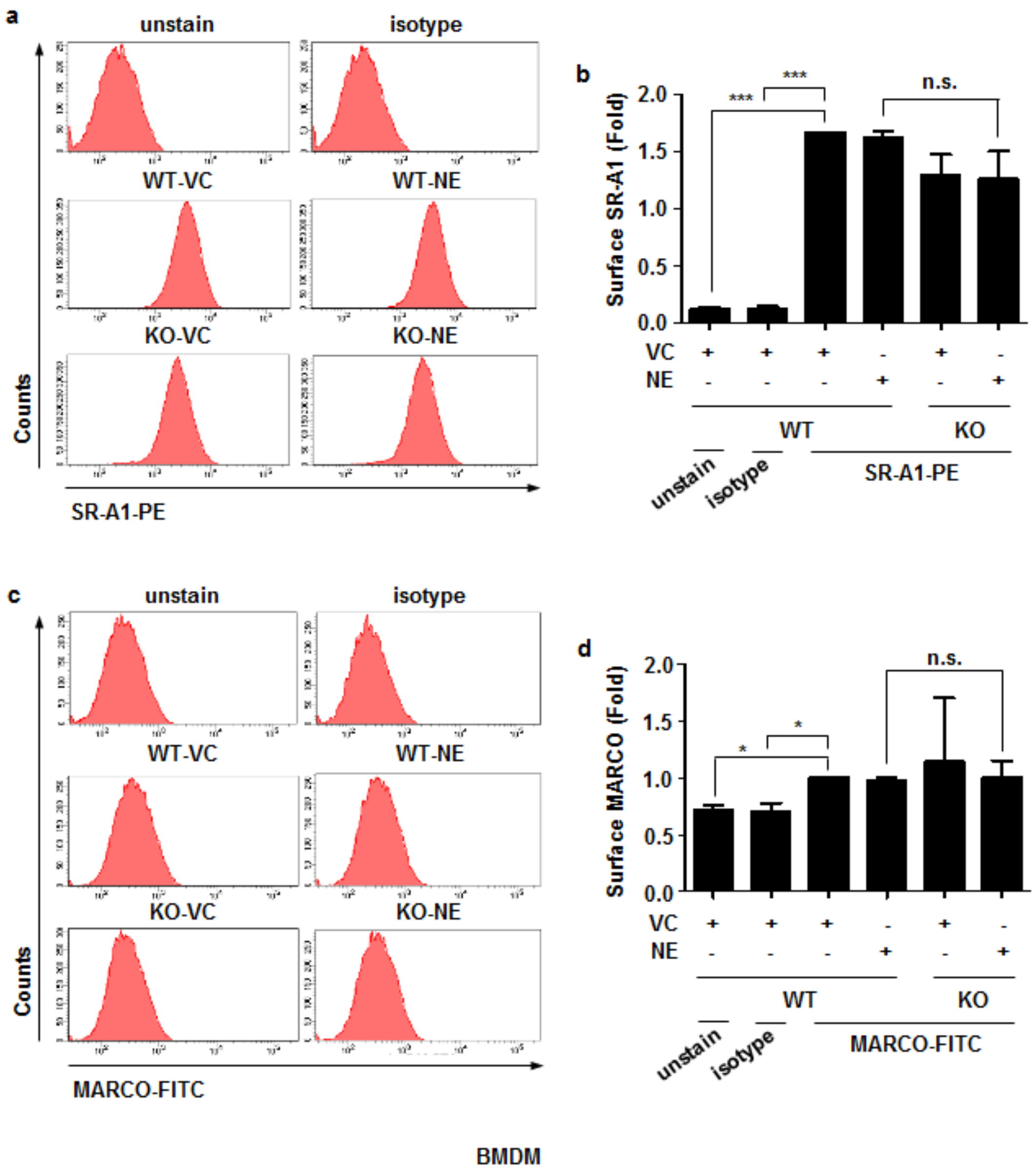

Supplementary Figure 4. *Crbn* KO did not affect the surface expression levels of scavenger receptors in elastase treated BMDM. WT and *Crbn* KO BMDM were stimulated with VC or NE (1U/mL) for 24 h. Cell surface expression of SR-A1 and MARCO was determined by flow cytometry. Results are shown as mean MFI histograms (a, c) (b, d) Data represent the mean ± SD of two separate experiments. * p<0.05 and *** p<0.001. n.s., non-significant; PE, phycoerythrin; FITC, fluorescein isothiocyanate.
